# Assembly of Ebola Virus Matrix Protein VP40 Is Regulated by Latch-Like Properties of N and C Terminal Tails

**DOI:** 10.1371/journal.pone.0039978

**Published:** 2012-07-05

**Authors:** Leslie P. Silva, Michael Vanzile, Sina Bavari, J. M. Javad Aman, David C. Schriemer

**Affiliations:** 1 Department of Biochemistry and Molecular Biology, University of Calgary, Calgary, Alberta, Canada; 2 United States Army Medical Research Institute of Infectious Diseases, Frederick, Maryland, United States of America; Universität Erlangen-Nürnberg, Germany

## Abstract

The matrix protein VP40 coordinates numerous functions in the viral life cycle of the Ebola virus. These range from the regulation of viral transcription to morphogenesis, packaging and budding of mature virions. Similar to the matrix proteins of other nonsegmented, negative-strand RNA viruses, VP40 proceeds through intermediate states of assembly (e.g. octamers) but it remains unclear how these intermediates are coordinated with the various stages of the life cycle. In this study, we investigate the molecular basis of synchronization as governed by VP40. Hydrogen/deuterium exchange mass spectrometry was used to follow induced structural and conformational changes in VP40. Together with computational modeling, we demonstrate that both extreme N and C terminal tail regions stabilize the monomeric state through a direct association. The tails appear to function as a latch, released upon a specific molecular trigger such as RNA ligation. We propose that triggered release of the tails permits the coordination of late-stage events in the viral life cycle, at the inner membrane of the host cell. Specifically, N-tail release exposes the L-domain motifs PTAP/PPEY to the transport and budding complexes, whereas triggered C-tail release could improve association with the site of budding.

## Introduction

The Ebola virus (EBOV) is an important human pathogen that causes severe haemorrhagic fever with a 50–90% mortality rate in humans [1]. Death is commonly due to the hypersecretion of numerous proinflammatory cytokines, chemokines, and growth factors [2], followed by breakdown of vascular epithelium and multiorgan failure [3]. The filoviruses are zoonotic pathogens and have been classified by the US Centres for Disease Control and Prevention as Category A bioterrorism agents. The development of detection methods, filovirus vaccines and therapeutics is therefore an important undertaking [4]. Progress has been made in recent years towards the development of candidate vaccines for EBOV prevention [5], [6] however the development of antiviral drugs and other post-exposure interventions has had mixed success. Warren and colleagues showed rhesus macaques treated with EBOV specific anti-senses targeting VP24 and VP35 protected them against lethal EBOV challenge [7]. Further, rhesus macaques treated with siRNA targeting three viral gene products (L polymerase, VP24 and VP35) were protected when given treatment regularly for 6 days after EBOV challenge, beginning 30 minutes from exposure to the virus [8]. These data suggest that interference with viral machinery could be an effective strategy for post-exposure treatment. However, it was subsequently shown that proteins encoded by EBOV (VP30, VP35, and VP40) act independently as suppressors of RNA silencing, indicating that the virus actively resists cellular RNAi during replication [9].

Additional insights into viral replication mechanisms are required in order to generate new vaccine candidates and therapies. A recent study demonstrated that inhibitors of host/viral-protein interactions may be effective [10], suggesting that new treatment options await a better understanding of viral mechanisms. As live virus experiments can only be conducted under Biosafety Level-4 conditions [11] the use of replication-defective vectors and virus-like particles (VLPs) is essential [12], [13]. EBOV has a compact 19 kb genome comprised of seven linear genes that encode for seven structural proteins [14], [15], expanding to nine with transcriptional editing [16]. Expression of the matrix protein VP40 is sufficient to generate VLP’s in a mammalian host that are remarkably indistinguishable from live virus, from a morphological standpoint [17]. Co-expression with viral glycoprotein GP and nucleocapsid protein NP generate VLP’s with improved viral egress [18], and serve as excellent models for studying later stages of viral replication (e.g. particle maturation, budding and release). The central role of VP40 in the life-cycle of the virus presents interesting opportunities for therapeutic intervention, but several questions remain regarding the molecular and structural properties underlying its function [19].

Matrix proteins such as VP40 are critical to the assembly of viruses and virus-like particles for several pathogens [20], [21], [22]. Such proteins interact with membranes in a hydrophobic and/or electrostatic manner and co-opt the host-cell membrane for final encapsulation [23]. EBOV VP40 forms a layer associated with the inner leaflet of the lipid bilayer [24], [25]. The L-domain of the N-terminal tail in VP40 (amino acids 1–52) represents a classical late-budding domain common among human pathogens, used in the recruitment of cellular machinery for endosomal sorting and transport (the ESCRT complexes). One candidate host protein interacting with the L-domain through its PTAP motif is Tsg101 [26], which may engage both ESCRT and ubiquitination machineries to drive budding and release at the plasma membrane. However, initial interactions with the inner membrane appear to be driven by the engagement of the C-terminal domain. VP40 is recruited to membrane microdomains involving C-terminal residues 309–317, proline 283 and proline 286 [24]. Interestingly, with the seven amino acids at the extreme C-terminus removed, a correlation between membrane engagement and the formation of intermediate assemblies of VP40 has been determined. VP40(31–319) spontaneously generated a hexameric ring-like form, whereas VP40(31–326) required either interaction with high concentration urea or liposomes to induce a similar hexameric state [25], [27]. It was noted that removal of the last seven residues does not adversely affect membrane association or recruitment of assembled VP40, but rather association may be improved by their removal [25]. It was proposed that residues 320–326 of the extreme C-terminus were therefore required to stabilize the association of the N and C terminal domains in a “closed” conformation in some fashion, even though this region was not observed in the crystal structure for the monomeric state.

X-ray diffraction data has been obtained from self-assembling VP40 truncations, in which the N-terminal tail and the entire C-terminal domain have been removed [28]. In the presence of short RNA strands VP40(55–194) forms an octamer, confirming that assembly occurs through the N-terminal domain. This logically requires the translocation of the C-terminal domain through a hinging motion, when comparing the octameric structure with the closed-form of the monomer [29]. This is supported by EM data of the hexameric state as well [25]. Both assemblies appear to share an identical dimer “building block” in which the N-terminal domains are antiparallel, but with different associations between dimers [27], [28], [30].

VP40 and the oligomerization process have been found to regulate viral transcription, packaging and particle formation, transport, membrane binding, as well as egress [31], [32], [33]. It remains an open question as to how the process of oligomerization can control so many disparate functions in the viral life-cycle. The extreme N and C terminal tails have been implicated, but their role remains speculative, in part as structural analyses used truncations rather than full-length protein. For example, structures including the N-terminal 43 amino acid tail (where the L-domain resides) are unavailable in either monomeric or oligomeric forms, suggesting some disorder in this region. It is therefore difficult to understand how assembly serves to regulate budding/viral egress through PTAP/PPxY recognition in the L-domain, if there is no observable structural transition in this region. Further, it remains an open question whether RNA-binding is an important step in higher-order assembly and packaging, or whether binding occurs only after such assemblies are formed. To address these questions, and to refine existing models of VP40 structure-function, the current study uses full-length VP40(1–326). Hydrogen/deuterium (H/D) exchange mass spectrometry was used to follow urea-induced monomer denaturation curves, in order to test for structural intermediates that may be accessed during assembly. The role of RNA in the assembly process was investigated in a similar fashion. We propose that both N and C tails function as a latch, preventing domain separation and assembly until recruitment of RNA, whereupon the latch is released. We suggest that this triggering provides a means of prioritizing RNA packaging and assembly ahead of tail-driven processes for viral transport, budding, and egress.

## Results

### Indexing VP40 Mass Map

**Figure 1:**
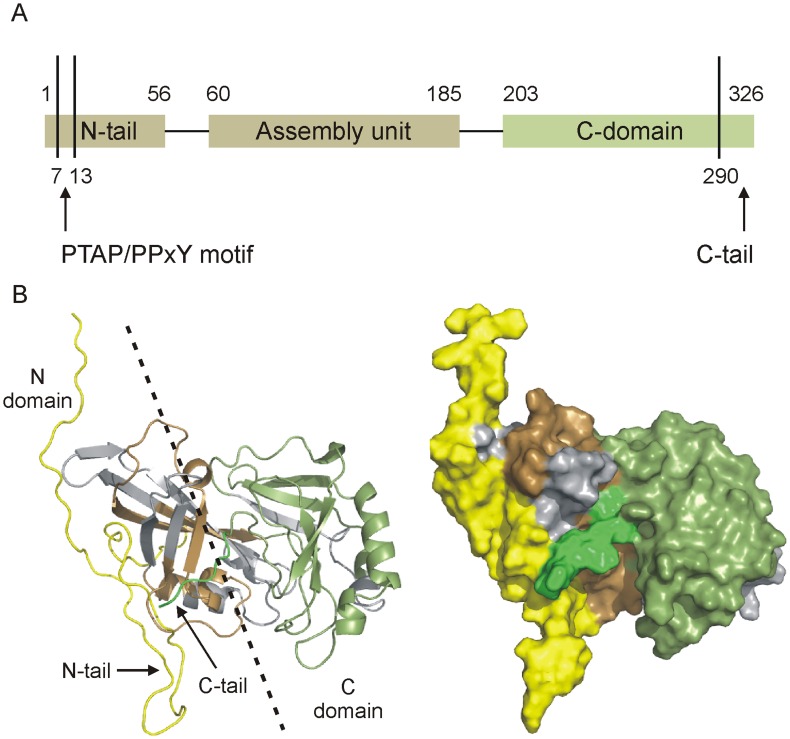
Structural representations of full length VP40 from Ebola virus Zaire. (A) Domain organization of VP40, reflecting the N-domain (tan) and the C-domain (olive). Regions of the N-tail contain a proline-rich motif known to interact with host WW protein modules, whereas the C-tail region is essential for membrane association and viral egress. (B) Cartoon and surface representations of VP40 with missing regions of structure modeled in a minimally biased manner. N-tail (1–44) and extreme C-tail (320–326) are shown in yellow and green, respectively. Domain coloring as in (A), with regions in grey representing missing sequence coverage.

In this study, mass shift experiments based on H/D exchange processes [34] were carried out using full length VP40(1–326), to test for transitional states of assembly and to probe the assembly of VP40. The first step was to develop a peptide mass map for tracking mass shifts at higher resolution. A two-minute pepsin digestion was found to be optimal, resulting in 32 high-intensity peptides, providing 75% sequence coverage. These peptides were used for all experiments in this study. Although coverage was not complete, we obtained full and redundant coverage of the N and C terminal tails. Lack of coverage is primarily in the N-terminal domain, which is only 52% complete This region is already known to self-assemble in VP40 truncations, thus reduced coverage was considered tolerable. The C-terminal domain is covered to 85% (Figure S1). Figure 1 outlines the domain organization for VP40 and displays the sequence coverage obtained in these experiments, mapped to a monomer structure that was augmented through modeling activities (see Materials and Methods). The sequence map can be found in Figure S1.

### Assessment of the VP40 Aggregation Status

**Figure 2:**
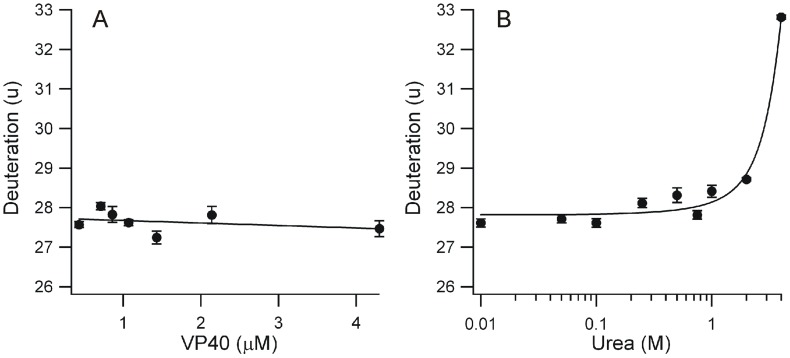
Stability analysis of monomeric VP40 at the protein level. (A) Effect of VP40 concentration on its aggregation status as determined with H/DX-MS. (B) Conformational stability of monomeric VP40 upon treatment with increasing urea concentration, also using H/DX-MS as a readout. Each datapoint represents the average of 3 replicates (±1 SD) using a composite of peptides as described in Materials and Methods.

Viral matrix proteins are a challenge to analyze biochemically as they have a propensity to nonspecifically aggregate, particularly at higher concentration. Prior to conducting denaturation studies, it was therefore necessary to ensure that the monomeric state was preserved over the course of urea treatment. Aggregation would likely alter H/D exchange levels, and thus prevent a determination of transitional states that may otherwise be observable. An SDS-PAGE of cross-linked monomeric VP40 did not show evidence of non-specific aggregation, even under 4 M urea treatment (Figure S2a). This was confirmed using H/DX-MS over a 10-fold concentration range for the protein, in the absence of urea. Here, an analysis of aggregation status was not possible from the intact protein as might normally be done using H/DX, as VP40 was found to irreversibly adsorb to chromatographic supports during work-up. Therefore a method was employed based on data generated using the digestion protocol, summing peptide data representative of maximal sequence coverage (see Materials and Methods). This alternative approach confirmed that aggregation, if it occurs, is below quantifiable levels (Figure 2a). It is noteworthy that under these conditions we see no evidence of hexamerization, as was seen with truncated VP40(31–319) in both 0 and 1 M urea [25]. All further H/DX experiments were performed using either 2.1 or 4.3 µM VP40, as these higher concentrations allowed for improved peptide detection and ease of data analysis.

### Denaturation Analysis of Monomeric VP40

**Figure 3:**
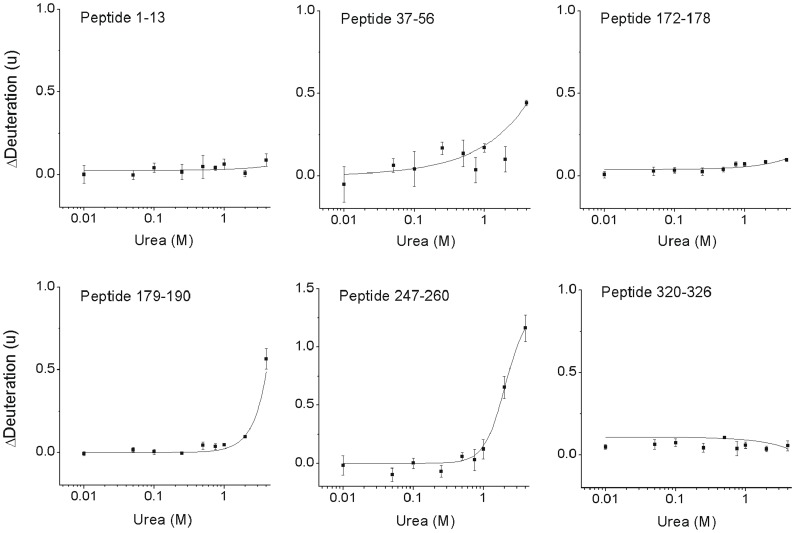
Higher resolution denaturation analysis of VP40. Graphs represent a selection of denaturation curves in key areas of structure (peptides 1–13, 37–56: N-tail; peptides 172–178, 179–190: N-domain and hinge; peptide 247–260: C-domain; peptide 320–326: extreme C-tail). Each datapoint represents the average of 3 replicates (±1 SD). Sigmoidal functions fit to the data for peptides 37–56, 172–178, 179–190 and 247–260. Straight line fit to the data for peptides 1–13 and 320–326.

The conformational stability of VP40 at 2.1 µM was then followed with H/DX as a function of urea concentration. The approach involving summed peptide data was used here as well, to provide a whole-protein evaluation of conformational transitions (Figure 2b). Only a single transition was observed, with an onset of approximately 2 M urea. (Note that reduced system performance at higher urea concentrations prevented analysis beyond 4 M denaturant.) While this whole protein view is useful to confirm the existence of large structural transitions, a higher-resolution analysis using the underlying peptide data may localize where such transitions occur, and uncover if other transitions are masked by the summed view. Individual curves were generated and inspected for all peptides. Only two classifications emerged based on a Tukey test: no significant conformational change at any urea concentration (21 peptides), and a conformational change beginning at approximately 1 M urea (11 peptides). Several regions of mechanistic significance are highlighted in Figure 3, with the remaining curves deposited in Figure S3. The L-domain region of VP40 containing the PTAP/PPEY motif is represented by peptide 1–13, which did not change its deuteration status over the entire urea concentration range. This may be due to full labeling at all concentrations, or it may indicate the absence of a conformational transition involving this portion of the N-tail. We note that labeling is extensive for this peptide but well below saturation. The full set of peptides in this region show a similar response (Figure S3b). The remainder of the N-tail, represented by peptide 37–56, was partially destabilized at high urea concentration. The C-tail, represented by peptide 320–326, also did not change deuteration levels over the entire urea concentration series. Thus, both N and C tail regions retain conformational stability even up to 4 M urea, indicating no significant change from the stable monomeric form.

**Figure 4:**
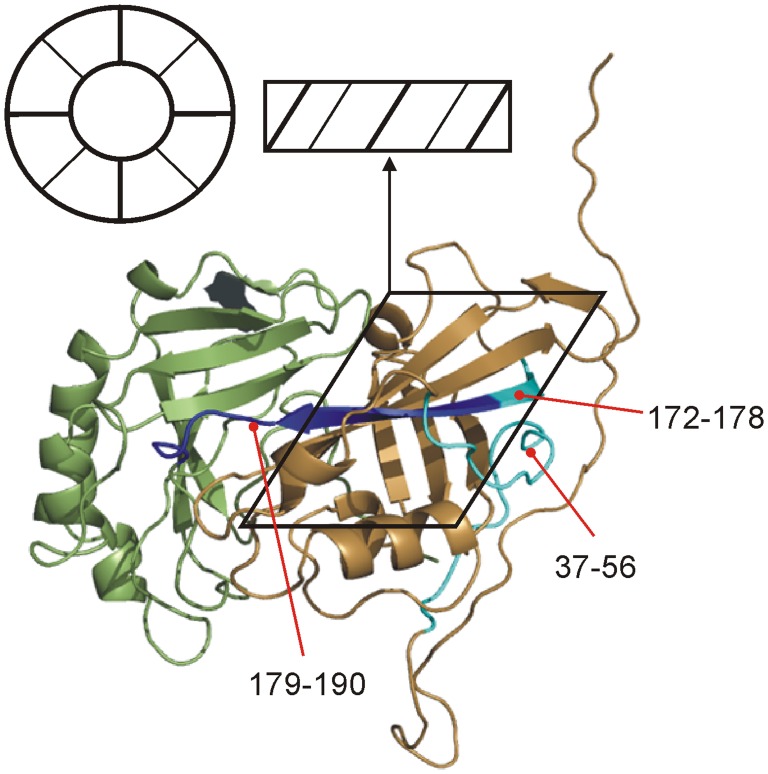
Effect of denaturation on N-tail and domain “hinge”. Weak destabilization of peptides 172–178 and 37–56 (cyan) suggest the partial exposure of an intradimer contact surface in the N-domain. Strong destabilization of peptide 179–190 suggests a urea-induced weakening of the hinge loop between C and N-domains. Black parallelogram roughly defines the oligomerizing unit. The structure is oriented 180° in the horizontal relative to Figure 1. Upon assembly into the octamer, the resulting ring would be on its side as shown by the arrow, highlighting the requirement for a large translocation of the C-domain prior to assembly.

Evidence supporting a modest distortion of peptide 37–56 in the N-tail is found in beta-strand 173–185, which defines an interaction surface for the C-terminal end of the N-tail (residues 46–56). Exposure of this surface upon conformational distortion of the N-tail should lead to a corresponding increase in deuteration. Peptide 172–178 in this region is indeed weakly destabilized (Figure 3), consistent with the overall resistance of beta-sheets to exchange. This beta-strand is noteworthy as it forms a portion of both the inter and intradimer contact in octameric assemblies [28]. The destabilization of peptide 179–190 in this region is 5 times greater than peptide 172–178. It is unlikely this is due to simple exposure of the remaining portion of the beta-strand. As the peptide also encompasses a loop connecting the C and N domains, it suggests instead a destabilizing of this “hinge” upon urea treatment (Figure 4). We note that the remaining urea-induced changes are found in the C domain flanking this hinge (e.g. peptide 191–208, Figure 3). While the largest perturbations are found here, they do not represent a full dissociation of the C-domain from the N-domain, *vide infra*. Overall, the denaturation study suggests a conformation that remains remarkably stable at high urea concentrations, except in the vicinity of this hinge.

### Analysis of Assembled VP40

When treated with 4 M urea and bulk RNA purified from *E.* *coli*, VP40 formed high-mass aggregates, as confirmed by SDS-PAGE and electron microscopy (data not shown). However, VP40 treated with trimeric RNA 5′UGA and 4 M urea as per previous structural studies induced assembly into ring-like structures [28], with a morphology consistent with that described for the octameric state [30]. Images consistent with an octameric state were evident in electron microscopy, with no higher order aggregation, and residual free monomer could not be detected with SDS-PAGE (Figure S2). This suggests near-quantitative conversion to an assembled state.

**Figure 5:**
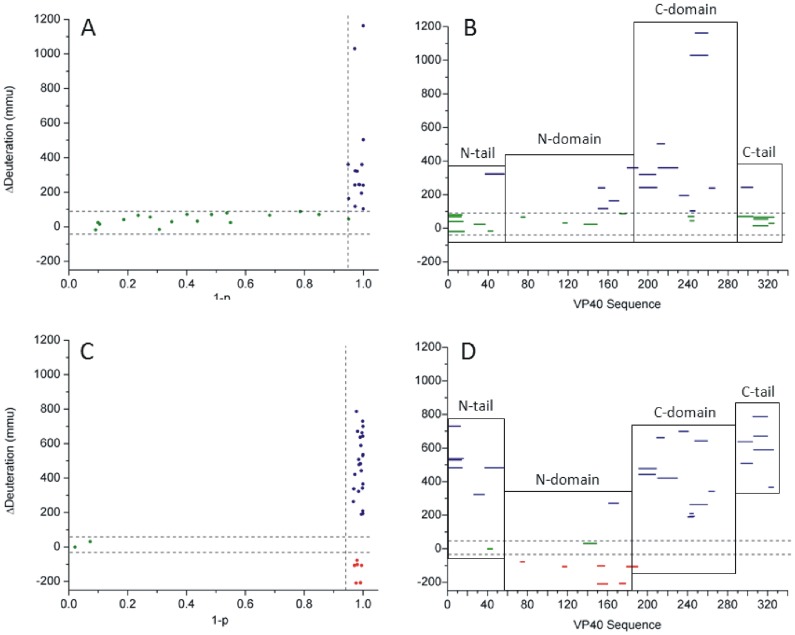
Influence of RNA binding on domain translocations. (A) Scatterplot of the H/DX-MS peptide data summarizing the effect of 4 M urea on the monomer, with (B) the corresponding sequence plot of the data. (C) Scatterplot of the H/DX-MS peptide data summarizing the effect of 5′UGA3′ addition to the urea-treated VP40 monomer, with (D) the corresponding sequence plot of the data. Significant destabilizations are noted in blue, stabilizations in red, and no observable change in green. Deuteration changes are presented in millimass units (mmu). Dashed lines demarcate regions of statistical significance (see Materials and Methods).

H/DX data was collected for this state and compared against the monomeric control treated with only 4 M urea. Paradoxically, although urea is used as a chaotrope in denaturation studies as above, it is also an inducer of assembly for truncated forms of VP40 [25], where assembly would lower deuteration rates. Under such conditions, perturbations of H/D exchange could be amplified relative to the control for assembly-induced destabilizations, and perhaps also at sites of assembly. The perturbations resulting from RNA addition and the attendant octamer assembly are shown in Figure 5. These are referenced to the urea-induced structural transitions of the monomer described above, specifically comparing the 0 and 4 M urea datasets. Summarizing the data for the urea treated of monomer, 47% of peptides demonstrated an increase in deuteration while the rest did not change (Figure 5a). Upon assembly in the presence of RNA, 75% of peptides demonstrated an increase in solvent accessibility, 19% demonstrated a decrease in solvent accessibility, and only 6% showed no change (Figure 5c). Corresponding plots mapping these changes against regions of structure are shown in Figure 5b,d.

**Figure 6:**
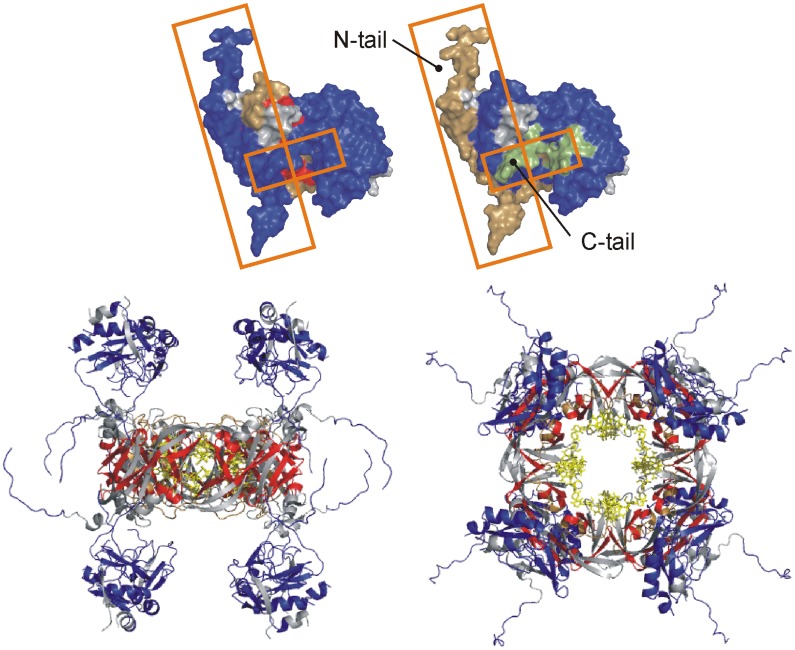
RNA-induced tail separation and assembly. Top structures: VP40 monomer model with full sets of H/DX data showing RNA-induced changes (left structure) and urea-induced changes (right structure). Boxes mark the N and C-tails that are denatured only upon RNA binding. Orientation and color scheme as in Figure 1. Bottom structures: octamer model in side and top views, with the H/DX data superimposed. Blue denotes deprotection and red protection. Grey regions are not represented in H/DX data, and yellow highlights the bound 5′UGA3′.

The addition of RNA generated a significant conformational change in the monomer. This is evidenced in the first place by protection from labeling in almost all peptides representing the oligomerizing N-domain. These peptides encompass both the inter- and intradimer interfaces created upon assembly. Although some degree of protection may directly arise from the binding of RNA to the N-domain, it is not possible to resolve this from assembly-induced reduction in labeling, and coverage in the areas known to bind RNA is poor. RNA binding triggers extensive destabilization in the C-domain, in all regions including the hinge. Thus, the destabilization of the C-domain by urea treatment alone (Figure 5b) does not represent a full disengaging of the C-domain from the N-domain; this appears to require RNA binding. Most notably, the ligation of RNA triggers an extensive denaturation of both tail regions. This is seen in the large positive increases in labeling for regions 1–56 and 290–326 (Figure 5d). These RNA-induced changes are mapped against the monomer structure and a model of the octameric assembly based on full length VP40, where the effect of RNA binding on the tail regions is readily observable (Figure 6).

## Discussion

In the molecular economy of viruses, proteins serve many roles. As a matrix protein and the most abundant protein of the Ebola virus, VP40 has been implicated in many stages of the viral life-cycle, particularly the later ones. Regulation of its conformational state is the proposed mechanism by which these diverse functions are invoked [19]. To explore the linkage between function and conformation, we applied H/DX-MS to full-length VP40, using denaturation analysis to add energy to the protein, in order to mimic the destabilization that might occur upon interaction with the membrane, host proteins or both. Our goal was to establish if full length protein exists in a metastable configuration of two domains, readily released and thus able to self-assemble. An additional goal was to determine how the extreme ends of both domains, each representing important interaction motifs, are influenced by assembly processes, given that the functions represented by these motifs are relegated to the sites of viral assembly and budding (i.e. lipid rafts in the host-cell membrane).

Here, we demonstrate that the full-length monomer is remarkably stable in the presence of high concentrations of urea and incapable of self-assembling, a finding that is unanticipated given that the crystal structure of the VP40(44–321) monomer shows a loose packing of the C and N domains connected by a flexible linker. VP40(31–326) was found to completely oligomerize at 4 M urea [25], which therefore indicates a role for the N-tail in maintaining stability. The removal of the C-tail (the last 7 amino acids) was shown in the same study to permit self-assembly around the N-domain, requiring only 1 M urea to induce complete assembly of VP40(31–319). Our structural model of the full-length monomer suggests that the additional resistance to denaturant arises from a latch-like association between the N-tail and C-tail. An accurate structural representation of these tails is not claimed, as the extreme N-tail would retain conformational freedom, however key features arising from this model are consistent with the higher resolution H/DX-MS data we present in this study. At elevated urea concentrations, only the hinge region of the monomer becomes destabilized – both tails retain stability and these are situated on the opposite face from the hinge.

In all modeling iterations, the N-tail and C-tails attained orthogonal orientations similar to that shown in Figure 1, the only difference being the insertion of the C-tail under or over the N-tail. We show that a specific molecular interaction is required in order to induce destabilization of the tails. Only upon binding of the RNA trimer are both tails strongly denatured (Figure 5d and Figure 6), which is consistent with their release from a latch-like orientation and the initiation of assembly. Previous findings have demonstrated that the presence of RNA alone is insufficient to induce assembly thus it appears that a partial destabilization of the monomer may have a role in establishing an interface for RNA recruitment and induction of an organized assembly process. A priming event of this nature may arise through interactions with the inner membrane, as VP40(31–319) in the presence of liposomes have been observed to drive a hexameric formation [25].

**Figure 7:**
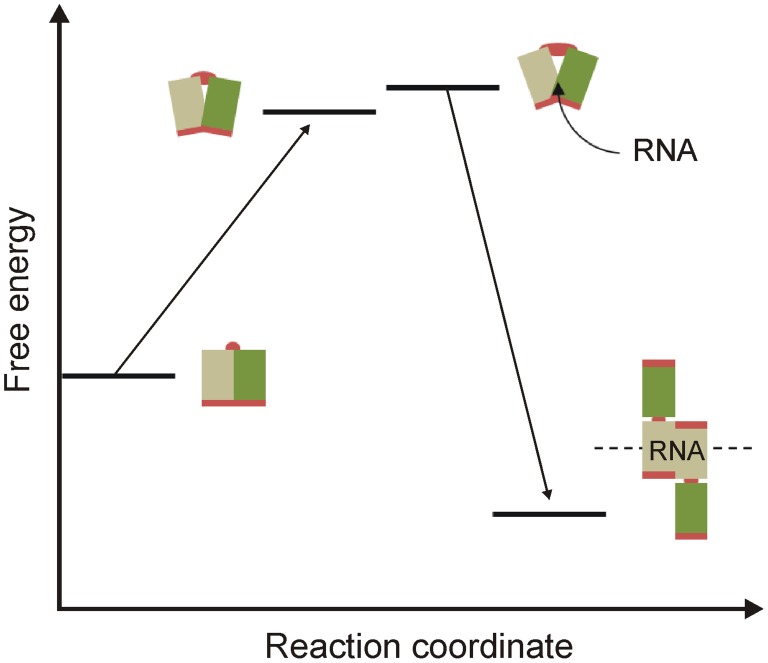
Free energy diagram for a proposed model of VP40 assembly. Destabilization of the monomer with urea mimics a native state where interactions between VP40 and the inner membrane induce a population of conformers, some of which are permissive of RNA binding. Tail release is triggered by RNA binding, followed in a concerted fashion by assembly and tail-mediated events in the viral life-cycle.

This is captured in a free-energy diagram for the assembly of VP40 octamers, which may have relevance for higher order assemblies (Figure 7). The initial contact of VP40 with the inner membrane, where assembly is orchestrated, is a process mediated by the C-domain. It was noted in our previous work that the extreme C-tail regulates coalescence with detergent-resistant membranes and eventual budding [24]. In the absence of this short tail, the C-domain interacts with detergent-soluble membranes but in a process that does not lead to budding and egress of VLPs. The domain may associate via electrostatic interactions through a positively-charged surface situated near the hinge, providing a distortion of the monomer sufficient to permit the recruitment of RNA for initiation of assembly. It is possible that RNA binding operates in a concerted fashion with recruitment to membrane microdomains, where late stages of assembly and budding occur, by releasing the C-tail and driving coalescence of VP40 from a diffusive membrane occupancy to the microdomains.

The peptide-level H/DX-MS data is of sufficient resolution to determine the effect of assembly upon the PTAP/PPxY motif, situated in the N-tail. In the structural model, this region of the N-tail is packed against the base of the assembly unit in the N-domain (Figure S4). The peptide containing this motif is highly destabilized upon assembly, suggesting its release and availability for recruitment of host proteins with WW domains. Key elements of the endosomal sorting complex involved in transport and vesicle generation in the host are coopted in the budding processes for many viruses, including Ebola virus [19]. Tsg101 interacts through the PTAP motif of VP40, and likely represents the entry point into recruitment of both the endosomal sorting and ubiquitination machineries for promotion of budding. Available structural data on PTAP/Tsg101 interactions show a deep binding groove in Tsg101 that would require a free tail for an effective interaction [35]. RNA-triggered assembly and release of this tail may represent a mechanism by which Tsg101 recruitment is relegated to late-stage events. Premature exposure of the tail could lead to ill-timed execution of budding and very likely affect the efficiency of VLP generation (Reynard, 2011 #162).

Finally, our study supports a structural model of the octameric intermediate in which the C-terminal domain does not participate in the ring assembly. The experimental process used in this work would denature regions of unprotected structure. Therefore the denatured C-domains observed upon assembly highlight their exclusion from the assembly unit. It is possible that the C-domain interacts somewhat more weakly with a region of the assembled N-domain structure, but our findings are consistent with the octameric model proposed in earlier work [28].

In summary, the conformational analysis used for tracking the induction of viral assembly intermediates reveals an unanticipated structural role for the C and N tails of Ebola virus matrix protein VP40. The tails lock a closed-monomer state until a combination of molecular triggers are invoked that are consistent with those known to occur at the inner membrane surface. The structural transitions observed in the tails suggests a mechanism by which late stages of viral assembly may be coordinated with viral packaging and release.

## Materials and Methods

All chemicals were purchased from Sigma Aldrich, St. Louis, MO, with the exception of LC solvents (HPLC grade water, acetonitrile, formic acid and trifluoroacetic acid), which were purchased from Thermo Fisher Scientific, Rockford, IL. Immobilized pepsin was purchased from Pierce (Thermo Fisher Scientific), Rockford, IL.

### Protein Production

Recombinant VP40 was expressed and purified in a manner similar to that described previously [30]. Briefly, wild-type Zaire Ebola virus VP40 (1–326) was expressed in E. coli strain BL21 (DE3) cells from a pET16B vector (N-terminal His tag). Expression was induced with 1 mM isopropyl-β-D-thiogalactopyranoside for 3 hours at 37°C. Cells were lysed by sonication in a buffer containing 50 mM Bicine, pH 9.3, 100 mM NaCl. The cleared supernatant was subsequently loaded onto an equilibrated Ni-affinity Sepharose column (Amersham Biosciences), and washed extensively. The protein was eluted in buffer containing 50 mM imidazole, and the His-tag removed with Factor Xa. After protease removal, the full-length protein was further purified by size exclusion chromatography on a Superdex 200 column equilibrated with 20 mM bicine, pH 9.3, 100 mM NaCl.

### Sample Preparation–denaturation and Aggregation Analysis

Recombinant full-length VP40 at a concentration of 2.1 µM was treated with urea at 10 different concentrations spanning 0–4 M. The protein was determined to be monomeric at this concentration in the absence of urea, based on H/D exchange measurements and crosslinking (see below). This was determined using a dilution series of VP40, over 0.15–4.3 µM. In all cases, urea treatment was conducted at room temperature for 20 minutes, in bicine buffer (20 mM bicine, 100 mM NaCl, pH 9.3) with 10 mM DTT.

### Sample Preparation–oligomerization

Full length VP40 at a concentration of 4.3 µM was incubated in 4 M urea and bicine buffer, with and without 5′UGA3′ RNA trimer (5 mg/ml) for 20 minutes at room temperature. The samples were then cross-linked with 5 mM Sulfo-EGS for 30 min. SDS-PAGE with coomassie brilliant blue staining was used to analyze reaction products.

### LC/MS – Mapping Sequence

VP40 (4.3 µM) was digested with immobilized pepsin for 2 minutes in a slurry of pH 2.3 (0.1 M glycine hydrochloride, GlyHCl). Peptides were separated with a 20 minute acetonitrile gradient in reverse-phased LC, using a prototype gradient pump (Upchurch Scientific, Oak Harbor, WA) and analyzed using an iterative IDA approach on a QSTAR Pulsar *i* Qq-TOF mass spectrometer (AB/Sciex, Concord, ON). Briefly, m/z space was binned into 150 Th windows to ensure full peptide selection per window. The MS/MS spectra were searched against the VP40 sequence using a local installation of Mascot 2.1, with conventional search criteria for pepsin digests.

### H/DX-MS

Deuteration data was measured on a per-peptide basis, using a bottom-up H/DX-MS strategy. Samples were labeled with 33% deuterated bicine buffer using an equilibrium labeling strategy, for 2 minutes. Labeling was quenched and simultaneously digested with the addition of a chilled slurry of immobilized pepsin in 0.1 M Gly-HCl at pH 2.3 for 2 minutes. Peptides were injected onto a chilled reversed phased LC system and separated in 20 minutes from injection. Peptide detection was achieved in TOF mode on a QSTAR Pulsar *i*. All samples were analyzed in triplicate.

### Data Analysis

Data were analyzed using Hydra v1.5 [36]. Deuterium levels were calculated using a fixed number of isotopes for each peptide and subtracting the theoretical mass of the undeuterated peptide using the same number of isotopes [36]. Identifying significantly mass-shifted peptides between two states (i.e. octameric vs. monomeric) was determined using criteria previously described [34]. Briefly, mass shifts must demonstrate a p-value less than 0.05 and exceed a threshold shift value based on a measurement of the noise in shift measurements. Data are shown in ΔD vs 1-p plots to permit assessment of data quality, and against their location in sequence space to identify which regions of the protein are affected by sample treatment conditions. To identify significant changes in the datasets arising from multiple sample states (i.e. multiple urea concentrations, multiple protein concentrations) a one way analysis of variance was employed in conjunction with a Tukey multiple comparison test [37] using Origin Pro v.8. H/DX dataplots representing the intact protein were generated by summing together the ΔD values from 20 peptides spanning the entire VP40 protein sequence, with minimal overlap.

### VP40 Structural Representations

Significant changes in deuteration were mapped to a model of VP40 based upon the VP40 (44–321) crystal structure PDB entry 1ES6 [29]. The full-length sequence was homology-modeled using 1ES6 as a template, in SWISS-MODEL [38]. This permitted the inclusion of missing minor loops within 44–321, but did not account for the missing N and C terminal tails. These were inserted manually, and the entire structure refined using the “relax” mode in Rosetta 3.1 [39]. The manual insertions were minimally biased, as they were placed in structure as disordered extensions. Two random orientations returned the same bend in the long N-tail upon relaxation, and close association between both N and C-tails. To develop a model of the octameric state, an initial octamer was created consisting of N-domains only, through the application of symmetry operations in the PDB entry 1H2D. C-domains were then added to this model from the 1ES6 PDB entry, with their position relative to the N-domain manually set. Part of the N-terminal tail (residues 44–68), was added to each monomer using internal coordinates from 1ES6, but with the relative orientation changed to a randomly selected ‘unhinged’ conformation to reflect H/DX labeling data. These additions were made to obey the octameric symmetry of the initial model. Manual coordinate manipulations were performed within the xLeap program of the Amber9 suite of programs and partial energy minimizations of manually manipulated regions were also performed in xLeap. All crystallographic water molecules were included. All structural images were rendered in the Pymol molecular graphics system, version 1.0r2 (Schrödinger, LLC).

### Electron Microscopy

Oligomers were generated as described previously [25], and 2 µl of sample was applied to a previously glow-discharged carbon-coated copper grid. Once the protein was adsorbed to the carbon, 5 µl of 1% uranyl acetate was applied as a negative stain, and excess liquid wicked off. Samples were photographed in a JEOL 1200 EXII electron microscope under low-dose conditions at a magnification of 40,000×.

## Supporting Information

Figure S1 **Figure S1 provides a sequence map for the matrix protein VP40.**
(DOC)

Figure S2 **Figure S2 provides additional biochemical evidence for the purity of the states of VP40 assembly studied in this work.**
(DOC)

Figure S3 **Figure S3 provides per-peptide mass shift data, completing the representative dataset in** **Figure 3** **.**
(DOC)

Figure S4 **Figure S4 shows a structural representation of full-length VP40, demonstrating the orientation of the WW domain relative to the rest of the structure.**
(DOC)
