# Supplementary material for: Assembly of Ebola Virus Matrix Protein VP40 Is Regulated by Latch-Like Properties of N and C Terminal Tails
Source: PLoS One. 2012 Jul 5;7(7):e39978. doi: 10.1371/journal.pone.0039978 (PMC3390324; doi:10.1371/journal.pone.0039978)
Supplement: Figure S1 — provides a sequence map for the matrix protein VP40. (DOC) [file pone.0039978.s001.doc]

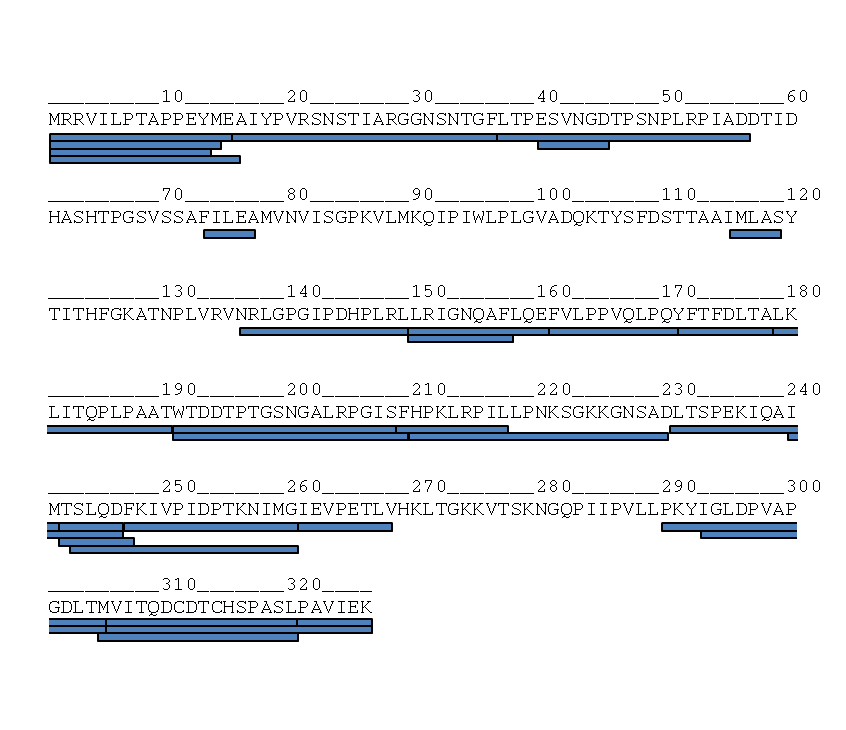


**Figure S1: Sequence coverage map of VP40.** Blue bars indicate the peptide size and position generated upon pepsin digestion in the H/DX-MS workflow. Sequence is from UniProt Q2PDK5 (VP40_EBOG4). See Figure 1, main text.
