# Supplementary material for: Assembly of Ebola Virus Matrix Protein VP40 Is Regulated by Latch-Like Properties of N and C Terminal Tails
Source: PLoS One. 2012 Jul 5;7(7):e39978. doi: 10.1371/journal.pone.0039978 (PMC3390324; doi:10.1371/journal.pone.0039978)
Supplement: Figure S2 — provides additional biochemical evidence for the purity of the states of VP40 assembly studied in this work. (DOC) [file pone.0039978.s002.doc]

**A B**

**1 2**

**
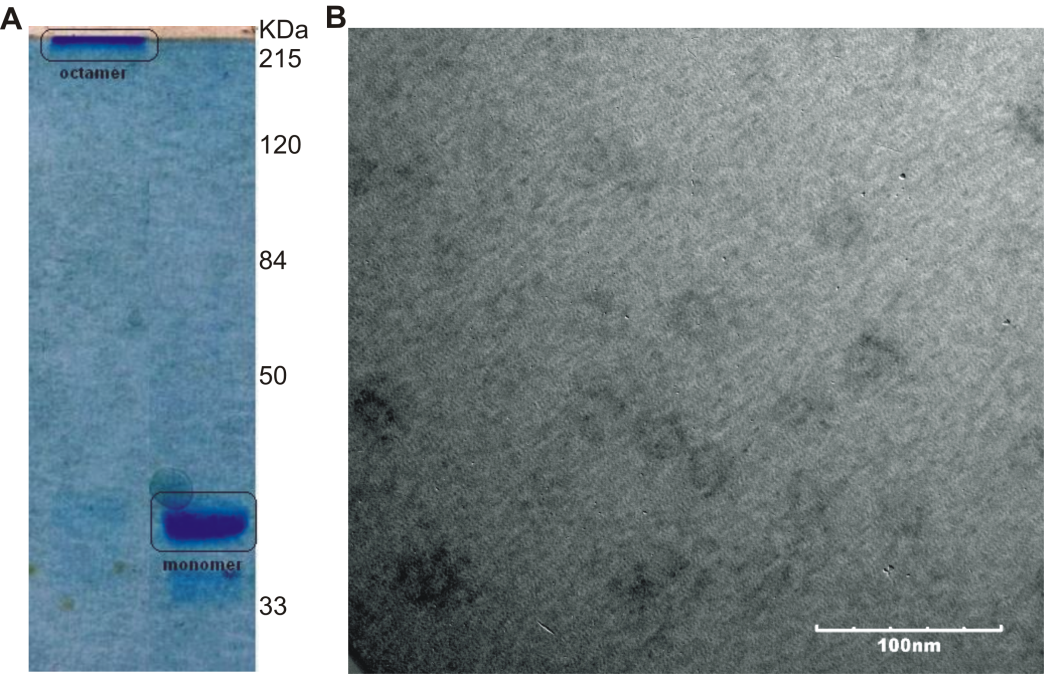

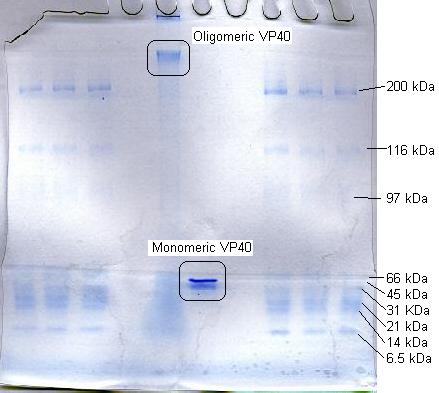
**

**Figure S2:** **Verification of VP40 octamer formation.** (A) SDS-PAGE gel stained with Coomassie brilliant blue, displaying 4.3µM VP40 treated with 4M urea and the 5′UGA3′ RNA trimer, crosslinked with 5mM Sulfo-EGS in bicine buffer (lane 1), and monomeric VP40 at 4.3µM in 4M urea (lane 2). (B) Negative-stain electron microscopic image of VP40 octamers, assembled from a concentration of 4.3µM VP40 in 4M urea and 5 mg/ml 5′UGA3′ RNA. See Results section in the main text (“assessment of VP40 aggregation status”), and Figure 2.
