# Supplementary material for: Assembly of Ebola Virus Matrix Protein VP40 Is Regulated by Latch-Like Properties of N and C Terminal Tails
Source: PLoS One. 2012 Jul 5;7(7):e39978. doi: 10.1371/journal.pone.0039978 (PMC3390324; doi:10.1371/journal.pone.0039978)
Supplement: Figure S3 — provides per-peptide mass shift data, completing the representative dataset in Figure 3 . (DOC) [file pone.0039978.s003.doc]

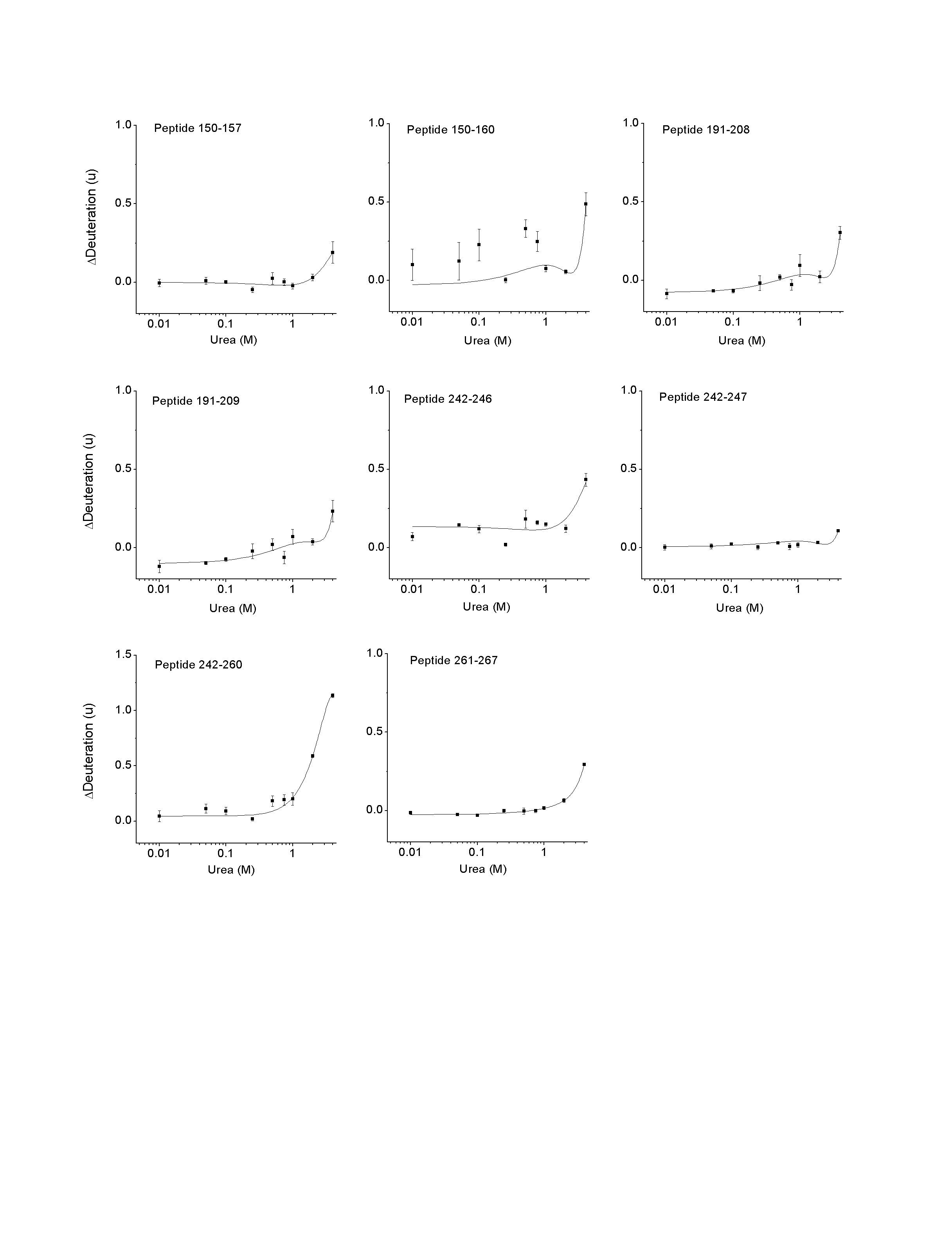


**Figure S3a:** **Denaturation curves for peptides showing a significant change in deuteration.** Each datapoint represents the average of 3 replicates (±1 SD) and the peptides are indicated within the individual figures. Third-order polynomials fit to the data for visualization purposes only. Together with the denaturation curves in Figure 3, this collection represents the remaining set of peptides that show a change based on the Tukey test. See Figure 3, main text.


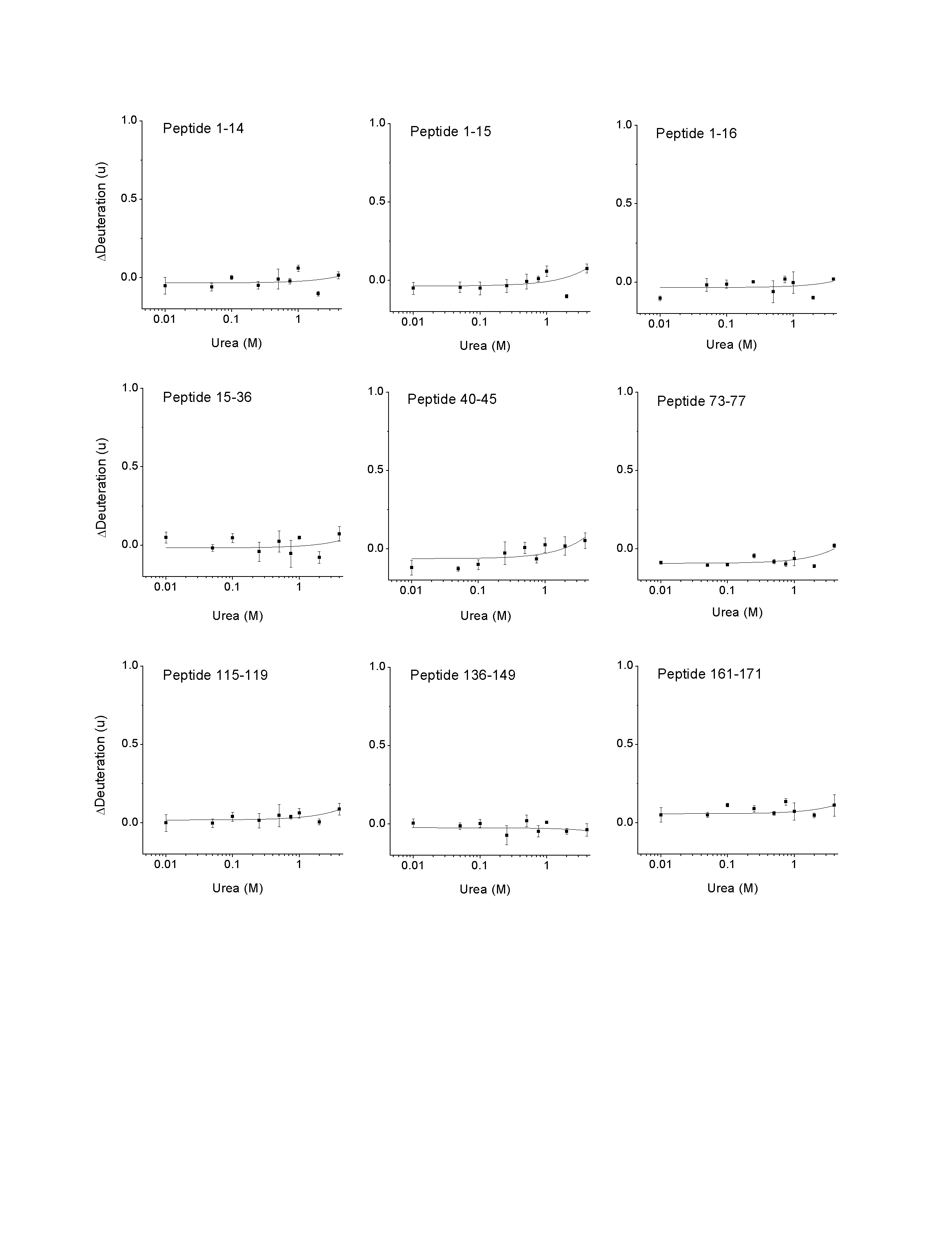


**Figure S3b:** **Denaturation curves for peptides showing no change in deuteration.** Each datapoint represents the average of 3 replicates (±1 SD) and the peptides are indicated within the individual figures. Linear interpolation applied to the data for visualization purposes. Together with the denaturation curves in Figure 3, this collection represents the remaining set of peptides for the N-domain that show no change in deuteration, based on the Tukey test. See Figure 3, main text.


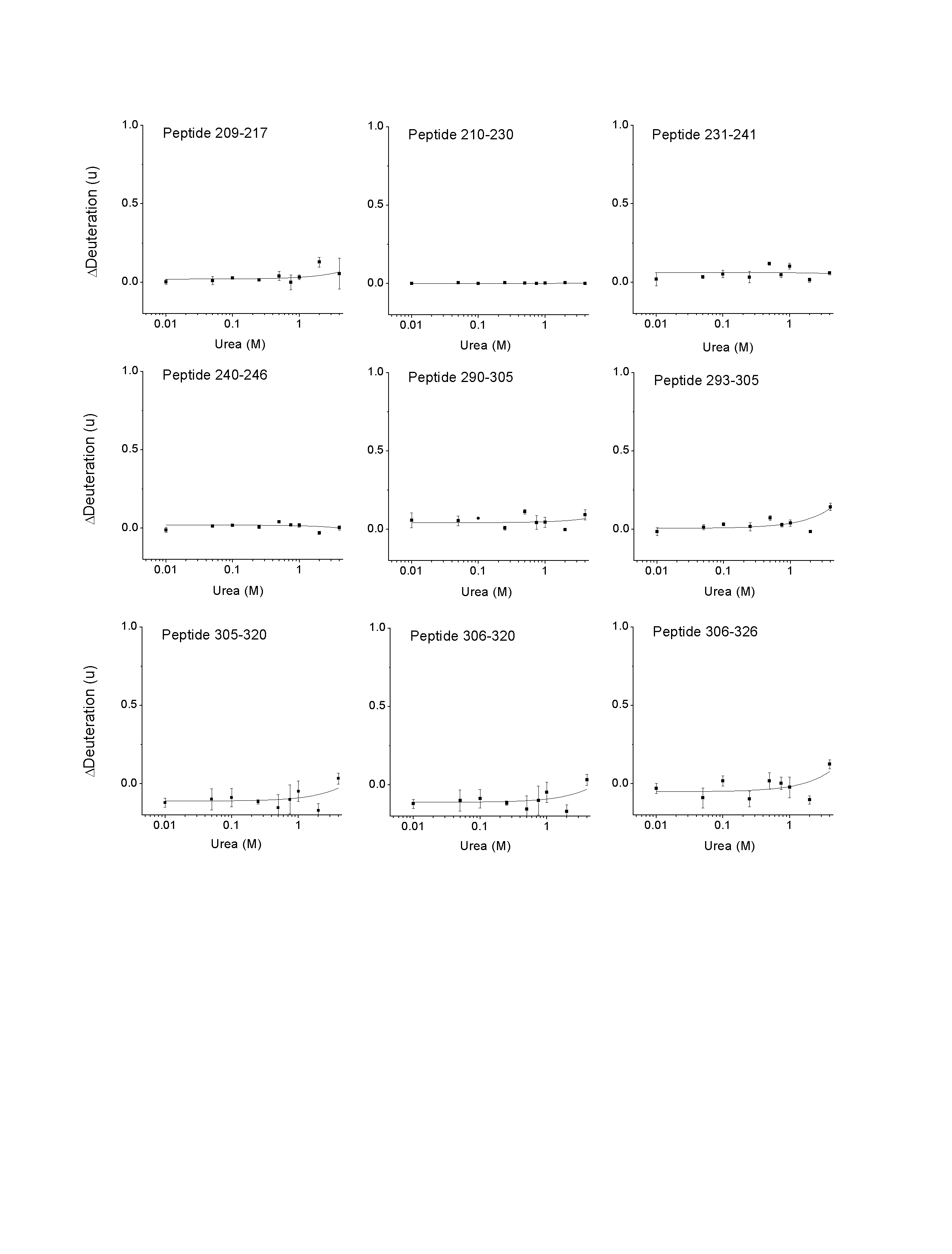


**Figure S3c:** **Denaturation curves for peptides showing no change in deuteration.** Each datapoint represents the average of 3 replicates (±1 SD) and the peptides are indicated within the individual figures. Linear interpolation applied to the data for visualization purposes. Together with the denaturation curves in Figure 3, this collection represents the remaining set of peptides for the C-domain that show no change in deuteration, based on the Tukey test. See Figure 3, main text.
