# Supplementary material for: Assembly of Ebola Virus Matrix Protein VP40 Is Regulated by Latch-Like Properties of N and C Terminal Tails
Source: PLoS One. 2012 Jul 5;7(7):e39978. doi: 10.1371/journal.pone.0039978 (PMC3390324; doi:10.1371/journal.pone.0039978)
Supplement: Figure S4 — shows a structural representation of full-length VP40, demonstrating the orientation of the WW domain relative to the rest of the structure. (DOC) [file pone.0039978.s004.doc]

**
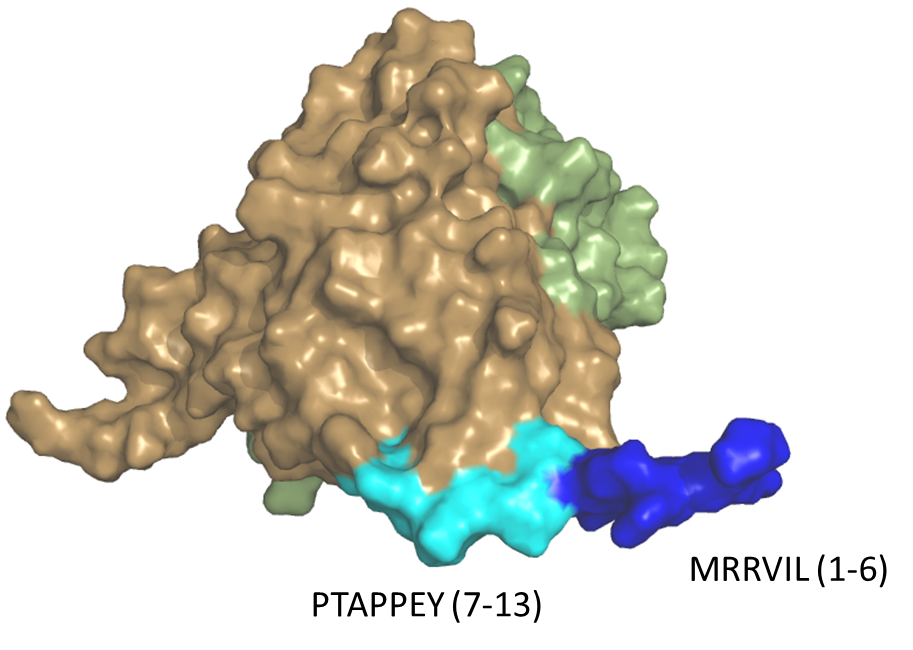
**

**Figure S4:** **Orientation of the overlapping WW motifs in the model of full length VP40.** Surface rendering of VP40 follows the color schemes as in the main text, with the PTAPPEY motif shown in cyan, in contact with the base of the N-domain assembly unit (in tan). The peptide detected in H/DX-MS (1-13) encompasses this motif plus the first 6 amino acids (in blue). These 6 amino acids likely do not change their deuteration profile upon VP40 assembly, as they are exposed throughout. Therefore, the deuteration change observed in peptide 1-13 upon assembly is attributed to the PTAPPEY motif. See Figure 5, main text.
